# Supplementary material for: Predictive factors for limited health literacy among persons with cirrhosis: A Swedish explorative cross-sectional study
Source: PLoS One. 2025 May 7;20(5):e0321780. doi: 10.1371/journal.pone.0321780 (PMC12058021; doi:10.1371/journal.pone.0321780)
Supplement: S2 File — S2 Table 1 reporting associations between patient characteristics and limited health literacy. S2 Table 2 reporting associations between cirrhosis related disease events and limited health literacy. (DOCX) [file pone.0321780.s002.docx]

**S2. Detailed logistic regression analyses of health literacy among patients with cirrhosis**

**S2 Table 1: Associations between patient characteristics and limited health literacy**

| **Predictor** | **Standard error** | **Risk Ratio**  **(95% Confidence Interval)** | **p-value** |
| --- | --- | --- | --- |
| **(Intercept)** | 0.0933 | 0.405(0.258-0.636) | <0.001 |
| **Age**  18-64 *vs* 65-85 | 0.2368 | 1.199(0.814-1.766) | 0.358 |
| **Gender**  Female *vs* male | 0.2330 | 1.141(0.764-1.702) | 0.519 |
| **Education**  Upper secondary school/university *vs* elementary school | 0.2917 | 1.493(1.018-2.189) | 0.040 |
| **Comorbidity**  No *vs* yes | 0.1452 | 0.635(0.405-0.994) | 0.047 |
| **Alcohol related liver disease**  No *vs* yes | 0.1775 | 0.838(0.553-1.269) | 0.404 |
| Goodness of fit (R^2^)=0.0523 | | | |

**S2 Table 2: Associations between cirrhosis related disease events and limited health literacy**

| **Predictor** | **Standard error** | **Risk Ratio**  **(95% Confidence Interval)** | **p-value** |
| --- | --- | --- | --- |
| **(Intercept)** | 0.0692 | 0.407(0.292-0.568) | <0.001 |
| **Work ability**  No *vs* yes | 0.4690 | 1.600(0.901-2.842) | 0.109 |
| **MELD-score**  ≤10 vs ≥11 | 0.2374 | 1.192(0.807-1.762) | 0.377 |
| **Covert hepatic encephalopathy**  No *vs* yes | 0.3188 | 1.544(1.031-2.314) | 0.035 |
| **Overt hepatic encephalopathy**  No *vs* yes | 0.2649 | 1.203(0.782-1.853) | 0.401 |
| **Ascites**  No *vs* yes | 0.2252 | 1.114(0.750-1.656) | 0.593 |
| **Oesophageal variceal bleeding**  No *vs* yes | 0.2434 | 1.049(0.665-1.653) | 0.838 |
| Goodness of fit (R^2^)=0.0697 | | | |
