# Supplementary material for: Predictive factors for limited health literacy among persons with cirrhosis: A Swedish explorative cross-sectional study
Source: PLoS One. 2025 May 7;20(5):e0321780. doi: 10.1371/journal.pone.0321780 (PMC12058021; doi:10.1371/journal.pone.0321780)
Supplement: Attachment — Submitted filename: Response letter_Hjorth and Forsberg.docx [file pone.0321780.s004.docx]

# Response to reviewers on manuscript ‘Predictive factors for limited health literacy among persons with cirrhosis: A Swedish explorative cross-sectional study’ (PONE-D-24-54410)

Dear Editor,

We thank you and the reviewer for constructive comments on our manuscript, entitled ‘Predictive factors for limited health literacy among persons with cirrhosis: A Swedish explorative cross-sectional study’ (PONE-D-24-54410), which have helped us to improve the text. In addition to the received comments, we have clarified that our analysis concerned *previous events* of hepatic encephalopathy and not *presence* of hepatic encephalopathy as we previously stated in the manuscript (page 9, row 162). We have reviewed all comments and suggestions thoroughly. Below we address them point by point. According to the instructions, the revised manuscript is uploaded in two examples 'Revised Manuscript with Track Changes' and ‘Manuscript’, without tracked changes. In line with the made changes, the supporting information have been revised, please see Appendix S1 and S2. We hope you will find our revisions satisfactory.

Kind regards,

The Authors

| EDITOR’S COMMENT | RESPONSE |
| --- | --- |
| If applicable, we recommend that you deposit your laboratory protocols in protocols.io to enhance the reproducibility of your results. Protocols.io assigns your protocol its own identifier (DOI) so that it can be cited independently in the future. For instructions see: [https://journals.plos.org/plosone/s/submission-guidelines#loc-laboratory-protocols](https://track.editorialmanager.com/CL0/https:%2F%2Fjournals.plos.org%2Fplosone%2Fs%2Fsubmission-guidelines%23loc-laboratory-protocols/1/010f019493ac2d53-59ae62be-bb69-483b-97ea-b562b1a285cf-000000/1ELSkGAxt4mAidvoOyo97erG0GDPPwJVwUjSCuSN66c=195). Additionally, PLOS ONE offers an option for publishing peer-reviewed Lab Protocol articles, which describe protocols hosted on protocols.io. Read more information on sharing protocols at [https://plos.org/protocols?utm_medium=editorial-email&utm_source=authorletters&utm_campaign=protocols](https://track.editorialmanager.com/CL0/https:%2F%2Fplos.org%2Fprotocols%3Futm_medium=editorial-email%26utm_source=authorletters%26utm_campaign=protocols/1/010f019493ac2d53-59ae62be-bb69-483b-97ea-b562b1a285cf-000000/Q-Nc24G0EspU1JOp9e3U_4_DtGnOmggdYGhu2X_a0uY=195). | Thank you for this opportunity. This manuscript refers to clinical patient data and do not include any laboratory data. Therefore we find this recommendation not applicable. |
|  |  |
| PLOS ONE FORMATTING COMMENTS |  |
| 1. Please ensure that your manuscript meets PLOS ONE's style requirements, including those for file naming. The PLOS ONE style templates can be found at  <https://journals.plos.org/plosone/s/file?id=wjVg/PLOSOne_formatting_sample_main_body.pdf> and  [https://journals.plos.org/plosone/s/file?id=ba62/PLOSOne_formatting_sample_title_authors_affiliations.pdf](https://track.editorialmanager.com/CL0/https:%2F%2Fjournals.plos.org%2Fplosone%2Fs%2Ffile%3Fid=ba62%2FPLOSOne_formatting_sample_title_authors_affiliations.pdf/1/010f019493ac2d53-59ae62be-bb69-483b-97ea-b562b1a285cf-000000/m7ym2qEurYqbps0Nw48TWS4DNtvcFsnJeT-RIxxCUz8=195) | According to PLOS ONE’s style requirements the following changes have been made:   - Symbol legends, line numbers and current addresses have been added to the front page. - In the revised manuscript, the in-text citation to Supplementary material now follow PLOS ONE’s requirements (page 10, rows 190-191; and page 11 rows 198-199). - Supporting information has been moved to the end of the manuscript. The text have been revised. Please see page 19, rows 409 to 416. - The supporting information files has been renamed, now reading S1 Appendix and S2 Appendix. |
| 2. In the online submission form, you indicated that [Data are available from Region Dalarna upon reasonable request (e-mail: [forsknings.utlamnande@regiondalarna.se](mailto:forsknings.utlamnande@regiondalarna.se)) provided that the data can be made available in accordance with applicable data protection and privacy regulations.].  All PLOS journals now require all data underlying the findings described in their manuscript to be freely available to other researchers, either 1. In a public repository, 2. Within the manuscript itself, or 3. Uploaded as supplementary information.  This policy applies to all data except where public deposition would breach compliance with the protocol approved by your research ethics board. If your data cannot be made publicly available for ethical or legal reasons (e.g., public availability would compromise patient privacy), please explain your reasons on resubmission and your exemption request will be escalated for approval. | Unfortunately, we are not able to share data publically due to restrictions in our approval from the Swedish Ethical Review Authority. The information in the online submission form has been revised, as follows:  ‘Data cannot be shared publicly because of restrictions by the Swedish Ethical Review Authority and the data register holders, as the data contains information that could compromise the privacy of research participants. Following approval by the Swedish Ethical Review Authority, the pseudonymized dataset analysed in this study are available from Region Dalarna ([forsknings.utlamnande@regiondalarna.se](mailto:forsknings.utlamnande@regiondalarna.se)).’ |
|  |  |
| REVIEWER’S COMMENTS |  |
| The authors are commended for conducting the first study of health literacy in a Swedish cirrhosis population. Health literacy is not adequately studied in patients with cirrhosis. In order for the manuscript to be strengthened the following need to be addressed. | Thank you for taking time and reviewing our manuscript. |
| 1. It is unclear if the patients were counseled by their hepatologists during the recruitment phase of the study spread over 24 months, introducing a bias in the assessment of HL. | We have added information about each participant regular hepatology consultations, which started at the time of cirrhosis diagnosis (page 5, row 94 to page 6, row 96). This study had a cross sectional design, collecting data at one single time point per patient, we therefore deem the risk of bias due to hepatology consultations being limited. |
| 2. The inclusion and exclusion criteria need to clearly stated. | Inclusion and exclusion criteria have been clarified with predefined comorbidities that resulted in exclusion. The information have been summarized in a new added table (Table 1), which is placed in the paragraph ‘Participant and setting’. Accordingly the text in the paragraph has been revised. Please see page 5, row 91 to page 6, row 106.  Due to the new added table, the number for the following tables in the manuscript have been revised (Page 10, row 184; page 10, row 190; and page 11, row 198) |
| 3. The methods particularly the Royal Free Hospital – Nutritional Prioritizing Tool (RFH-NPT) section need to be revised for clarity. A correlation between the Royal Free Hospital – Nutritional Prioritizing Tool (RFH-NPT), and HL is not linear, multiple confounders may impact the association, and needs to be justified. | After consideration we have decided to exclude RFH-NPT form the analysis. Consequently, we agree there is a high risk for confounders for this factor. This change has resulted in the following adjustment in the manuscript:   - The paragraph that described the RFH-NPT has been removed from the data collection section (page 8, rows 143-151). - Risk for malnutrition has been removed from the statistical analysis section (page 9, rows 163-164). - Risk ratio and confidence intervals for the second generalized logistic regression model have been adjusted in line with the new analysis (page 11, row 196 and Table 4). - The new analysis revealed a 54 % higher risk for limited health literacy following covert hepatic encephalopathy, instead of previously reported 70 %, (page 11, row 204). - Reference 28 has been removed, which is visible in the manuscript without track changes. |
| 4. Additionally, 70% higher risk of limited health literacy was exhibited in patients with covert hepatic encephalopathy, indicating a significant gap in the management of hepatic encephalopathy despite their symptoms. | We agree that there is a gap in the management of hepatic encephalopathy. Without systematic screening for hepatic encephalopathy, the symptom may not be identified, which is one of our important reported findings in this study. We have revised the text to clarify the problematic situation without screening routines for hepatic encephalopathy (page 12, row 206-207). |
| 5. It will be interesting to know if the same pattern pf limited HL was shown by patients with other GI disease. | According to Kaps et al 2022 (Reference 12). Health literacy in patients with cirrhosis is reported to be lower than for patients with gastrointestinal cancer or inflammatory bowel disease. In line with this comment, we have elaborated the text for clarification. Please, see page 13, rows 234-236. |
